# Diphtheria: Its Prevention and Control

**Published:** 1914-10

**Authors:** 


**Diphtheria: Its Prevention and Control.** By J. W. Schereschewsky, U. S. Public Health Service, Supplement No. 14 to the Public Health Reports, April 17, 1914. Price 5 cents.

This is a comprehensive up-to-date review of this subject and should be in the hands of every sanitary inspector, nurse and teacher to all of whom as well as to the physician it will give a clear understanding of the modern way of controlling this disease.—C. G. L-W.

